# FF-Net: Feature-Fusion-Based Network for Semantic Segmentation of 3D Plant Point Cloud

**DOI:** 10.3390/plants12091867

**Published:** 2023-05-01

**Authors:** Xindong Guo, Yu Sun, Hua Yang

**Affiliations:** 1 College of Information Science and Engineering, Shanxi Agricultural University, Jinzhong 030801, China; gxd@sxau.edu.cn (X.G.); sunyu@sxau.edu.cn (Y.S.); 2 College of Computer Science and Technology, North University of China, Taiyuan 030051, China

**Keywords:** plant phenotype, point cloud, semantic segmentation, feature fusion

## Abstract

Semantic segmentation of 3D point clouds has played an important role in the field of plant phenotyping in recent years. However, existing methods need to down-sample the point cloud to a relatively small size when processing large-scale plant point clouds, which contain more than hundreds of thousands of points, which fails to take full advantage of the high-resolution of advanced scanning devices. To address this issue, we propose a feature-fusion-based method called FF-Net, which consists of two branches, namely the voxel-branch and the point-branch. In particular, the voxel-branch partitions a point cloud into voxels and then employs sparse 3D convolution to learn the context features, and the point-branch learns the point features within a voxel to preserve the detailed point information. Finally, an attention-based module was designed to fuse the two branch features to produce the final segmentation. We conducted extensive experiments on two large plant point clouds (maize and tomato), and the results showed that our method outperformed three commonly used models on both datasets and achieved the best mIoU of 80.95% on the maize dataset and 86.65% on the tomato dataset. Extensive cross-validation experiments were performed to evaluate the generalization ability of the models, and our method achieved promising segmentation results. In addition, the drawbacks of the proposed method were analyzed, and the directions for future works are given.

## 1. Introduction

Plants, which are an important food source and a significant part of the ecological environment, are inextricably linked to the survival of humans [1]. To meet the demand for increasing grain production, breeding experts need to adopt efficient breeding programs to breed high-yielding and high-quality crop varieties [2]. A high-throughput number of plant phenotyping datasets can help researchers analyze and track the growth of plants, which is a great help to plant breeding [3]. However, compared to high-throughput measurements of crop gene sequences, traditional plant phenotyping methods based on artificial measurements are usually inefficient, which limits the process of modern agricultural intelligent breeding. In addition, measuring plant phenotypes manually may lead to inaccurate results, and direct contact with the plants would cause irreversible damage to the plants [4]. Hence, an automatic high-throughput plant phenotyping technique has become a research point in the field of modern digital agriculture.

The plant phenotype is a comprehensive assessment of the complex plant characteristics that we observe, including morphological parameters, traits, physiology, etc. For most plants, leaves, which are the main component of photosynthesis, take up the largest proportion of all organs [2,5]. Monitoring and analyzing phenotyping parameters such as the shape, color, and size of plant leaves in real-time could help to detect pests and diseases, predict crop yields, and select high-quality crop varieties. Furthermore, plant leaves are more regular than other organs with complex structures, and it is easy to obtain samples, so plant leaves’ phenotype is the most-essential task in plant physiology research. Therefore, an automatic, high-throughput, and accurate plant leaf segmentation method is of primary importance for downstream research tasks [6].

With the development of computer vision technology, image processing has been widely adopted in the field of modern digital agriculture [7]. Many image-based phenotyping methods, which are low-cost, non-invasive, and high-throughput, have been proposed to acquire detailed and particular plant traits [8]. Mao et al. [9] proposed an adaptive segmentation method of crop disease imaging based on the fuzzy C-mean clustering algorithm (FCM), which takes as the input the gray pixels and the mean of the neighborhood of pixels. Li et al. [10] designed a co-segmentation algorithm based on the optimization model of the Markov random field to generate a universal and accurate image segmentation of cotton leaves under natural lighting conditions. Singh et al. [11] proposed an algorithm for an image segmentation technique that is used for the automatic detection and classification of plant leaf diseases. Xie et al. [12] converted tobacco images into the Lab color space and then used support vector machine (SVM) to recognize and count tobacco plants. These methods could significantly improve the efficiency of plant phenotyping in a non-destructive way.

However, since plants usually have complex structures and occlusions exist in the leaves, these image-based methods still have some limitations in plant leaf segmentation. To address this issue, many researchers have introduced 3D point clouds into plant phenotyping [13,14,15,16]. On the other hand, with the rapid development of 3D sensing techniques, the way to obtain 3D data becomes less expensive and easier, such as 3D laser scanners, time of flight cameras, and light detection and ranging (LiDAR) [17,18,19]. Mortensen et al. [20] proposed a cluster-based method for segmenting lettuce in color 3D point clouds and estimating the fresh weight. Jin et al. [21] proposed a median-normalized vector growth (MNVG) algorithm to segment stems and leaves, after which the phenotypic traits of the leaf and stem were extracted. Hui et al. [22] used a multi-view stereo (MVS) approach to quantify and evaluate the canopy structure of plant populations and monitor the growth and development from the seedling to the fruiting stage. Itakura et al. [23] designed a method that automatically segmented 3D models constructed from scenes taken from different positions for the leaf areas and inclination angles. Jin et al. [24] proposed a voxel-based convolutional neural network (VCNN) to segment maize stem and leaf, and the results outperformed traditional methods.

Although the above-mentioned methods are effective for the segmentation of plant 3D point clouds, there still exist two main drawbacks of these methods: (1) traditional methods are not robust for plants in different stages owing to the variable sizes; (2) learning-based methods using only one representation of 3D point clouds cannot explore the point traits thoroughly, which limits the accuracy and generalization ability. The objective of this work was to address these two deficiencies in the segmentation of plant 3D point clouds. We evaluated the recently popular learning-based methods on plant 3D point cloud datasets (i.e., maize and tomato) and propose an adaptive voxel-based approach to extract local region features. Then, a feature fusion method based on an attention mechanism is proposed to coalesce pointwise features and voxelwise features.

In summary, the contributions of this work are as follows:

- To the best of our knowledge, this is the first work that uses a multi-representation of 3D point clouds to segment the leaves and stems of plants.
- An adaptive voxel-based method to partition plant 3D point clouds more evenly is proposed, then a sparse 3D convolution is introduced to accelerate the efficiency.
- An attention-based feature fusion method to merge point features from different modules is proposed, which may contribute to future developments of plant 3D point cloud segmentation.

## 2. Results

In this section, we first provide the detailed experimental setting and evaluation metrics and then report the results on two plant datasets, namely maize and tomato. Furthermore, a cross-validation experiment was constructed, in which we trained the models on the maize dataset and tested on the tomato dataset, and vice versa, to validate the generalization ability.

### 2.1. Experimental Setup

We compared our feature-fusion-based network with three popular learning-based methods (i.e., PointNet [25], PointNet++2017PointNet2, and DGCNN [26]) on the maize and tomato datasets. All networks were trained with Adam with a learning rate of 0.001 for 200 epochs on an Nvidia Geforce 3090 GPU. It is worth noting that our proposed FF-Net took as the input the entire plant point cloud without down-sampling, while the other three competitors have to down-sample the points as the memory cost is so large that an overflow occurs. Therefore, the batch size of FF-Net was set to 4, while the other three were 32.

### 2.2. Evaluation Metrics

For a fair comparison, we used the same strategy to train all models and report the per-class Intersection over Union (IoU) [25] and the mean IoU (mIoU) [25] over all classes. The IoU can be formulated as

(1) $$IoU_{i}=\frac{TP_{i}}{TP_{i}+FN_{i}+FP_{i}’}$$

where *TP* (true positive) denotes that the positive class is predicted as the positive class, *FN* (false negative) indicates that the positive class is predicted as the negative class, and *FP* (false positive) means that the negative class is predicted as the positive class.

The mean intersection over union (mIoU) is the average IoU of all classes, which can be formulated as

(2) $$mIoU=\frac{1}{N}\sum_{i=1}^{N}IoU_{i}.$$

### 2.3. Results on Maize Dataset

Results: Table 1 presents the semantic segmentation results on the maize datasets, from which we can observe that all methods achieved a high IoU (>95%) for the ground category and the second-highest IoU (>80%) for the leaf class. However, PointNet [25] almost failed to recognize the stem, which is a relatively small class with few points. As a result, PointNet [25] only achieved a 3.4% IoU for the leaf class. PointNet++ [27] and DGCNN [26] obtained a relatively small IoU (<40%) for the stem class, which mostly confounded stems with leaves. Our proposed method outperformed all other methods for both classes’ IoU and mIoU. Specifically, our proposed method achieved about a 20% performance gain over PointNet [25], 7% over PointNet++ [27], and 5% over DGCNN [26]. In addition, our proposed method outperformed the other methods for the stem class by a large margin (>15%).

**Figure 1:**
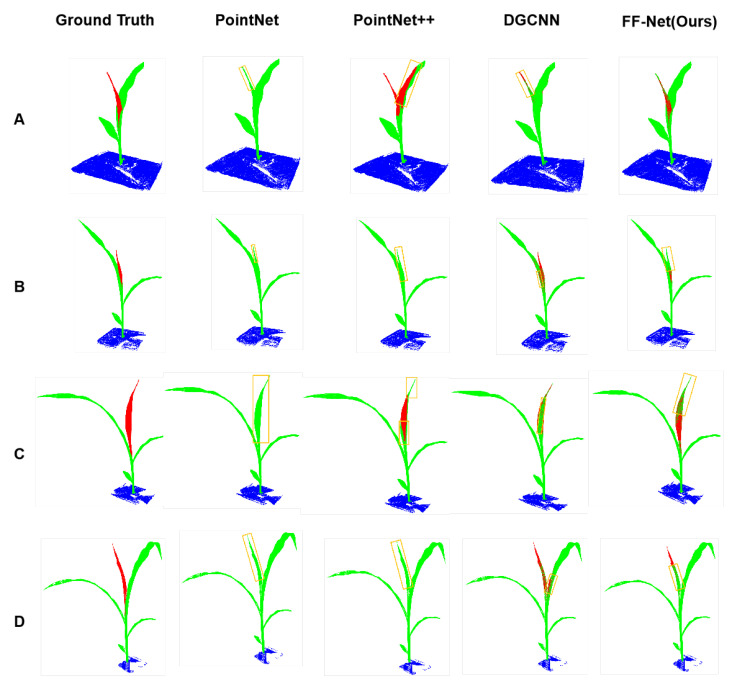
Semantic segmentation comparison on maize point cloud between our proposed method and other competitors. Row (**A**–**D**) indicates the maize point cloud captured at different stages.

Visualization: It can be seen from Figure 1 that PointNet [25] hardly identified the stems and recognized them as leaves (falling into the yellow boxes). Benefiting from the hierarchical feature extraction, PointNet++ [27] achieved some improvement in recognizing leaves, while still misidentifying some leaves as stems (e.g., Row A of PointNet++ [27]). It is worth noting that, in Rows B and D, both PointNet [25] and PointNet++ [27] misidentified the stems as leaves. The reason may be that the stems and leaves of these maize plants have very similar properties. By contrast, DGCNN [26] can identify the stems as shown in Rows B and D. As we can see from Figure 1, DGCNN almost recognized all stems, but still had some misidentification of the petiole (e.g., Rows B and D of DGCNN [26]). There existed another interesting phenomenon, where DGCNN misrecognized the outline of the leaf as the stem, as shown in Row C of Figure 1. From the last column of Figure 1, we can see that our proposed method, the FF-Net, produced the most-similar segmentation results as the ground truth. Nevertheless, our method still missed some part of the stem when recognizing, as shown in Rows B and D.

### 2.4. Results on Tomato Dataset

Results: The semantic segmentation results of tomatoes are shown in Table 2, from which we can see that all methods obtained a relatively high IoU for the ground (>99%) and leaf (>90%) category. However, PointNet [25] still struggled with the stem class and obtained a 28.35% IoU, which was about 25% higher than the prediction on the maize datasets before. PointNet++ [27] achieved a 48.78% IoU for the stem class, which was about 13% higher than the prediction for the maize datasets. DGCNN [26] obtained a 56.73% IoU for the stem class, which was 18% higher than the prediction for the maize datasets. As can be seen from Table 2, our proposed method consistently outperformed other methods in all three classes.

**Figure 2:**
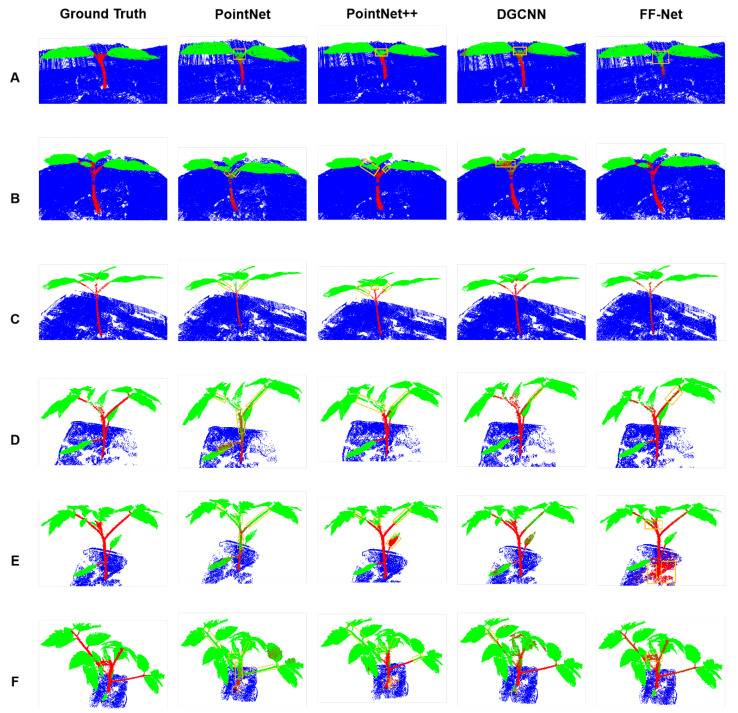
Semantic segmentation comparison on the tomato point cloud between our proposed method and other competitors. Row (**A**–**F**) indicates the tomato point cloud captured at different stages.

Visualization: Figure 2 presents the semantic segmentation results of the methods above on the tomato dataset. The parts of the misrecognition and missed recognition are outlined with yellow boxes. As we can see from Figure 2, PointNet [25] can recognize most of the stems that are far away from the leaves, as these stems have significantly different traits from the stems near the leaves. However, the stems that are close to the leaves have similar traits as the leaves, and it was a challenging task for PointNet to distinguish them. PointNet++ [27] performed better than PointNet [25] due to its hierarchical feature extraction mechanism. As shown in Figure 2, PointNet++ [27] identified more details of the stems than PointNet [25] (Rows D, E, and F). Nonetheless, there also existed some misidentifications of leaves for PointNet++ [27], e.g., Row E of PointNet++ [27], where a leaf in the middle of the plant was misidentified as the stem. We also found an interesting phenomenon that both PointNet and PointNet++ [27] hardly recognized stems surrounded by leaves, such as the stems on top of the plants in Rows D, E, and F. As for DGCNN [26], we can see that most of the stems were identified, even the hardest stems surrounded by many leaves. As shown in the rightmost column of Figure 2, our proposed method recognized most outlines of the stems, while still misidentifying some points of the stem as the leaf class. Even so, our method performed better than all other competitors in recognizing the details of the stems, as shown in Rows D, E, and F. It is worth noting that a part of the ground was misidentified as the stem, as shown in the last column of Row E in Figure 2, which caused the IoU value of the stem class to decrease.

### 2.5. Results of Cross-Validation

To evaluate the generalization ability of the model, we conducted extensive cross-validation experiments on both plant datasets. First, we studied the case when the models were trained on the tomato dataset and tested on the maize dataset. Then, we trained the models on the maize dataset and tested them on the tomato dataset. For a fair comparison, we adopted the same parameter settings as the aforementioned experiments and report the same metrics, namely class IoU and mean IoU. The details are introduced in the following sections.

#### 2.5.1. Training on Tomato and Testing on Maize

Results: Table 3 shows the segmentation results for being trained on the tomato dataset and evaluated on the maize dataset. Because the ground in both datasets had similar traits, all models maintained a high IoU for the ground class (>95%). As for the leaf class, all models obtained a relatively high IoU (>80%), except for PointNet. However, all models failed to recognize the stem class, especially PointNet and DGCNN [26], which only obtained IoUs of 0.35% and 0.69% for the stem class, respectively. Compared with the segmentation results of the stems being trained and evaluated on the same category of the maize dataset, the segmentation results for the stems for PointNet [25], PointNet++ [27], DGCNN [26], and FF-Net, which were trained on the tomato dataset and evaluated on the maize dataset, were down by 90%, 92%, 98%, and 87%. Our method achieved the smallest reduction in the IoU for the stem class when generalizing from the tomato dataset to the maize dataset.

**Figure 3:**
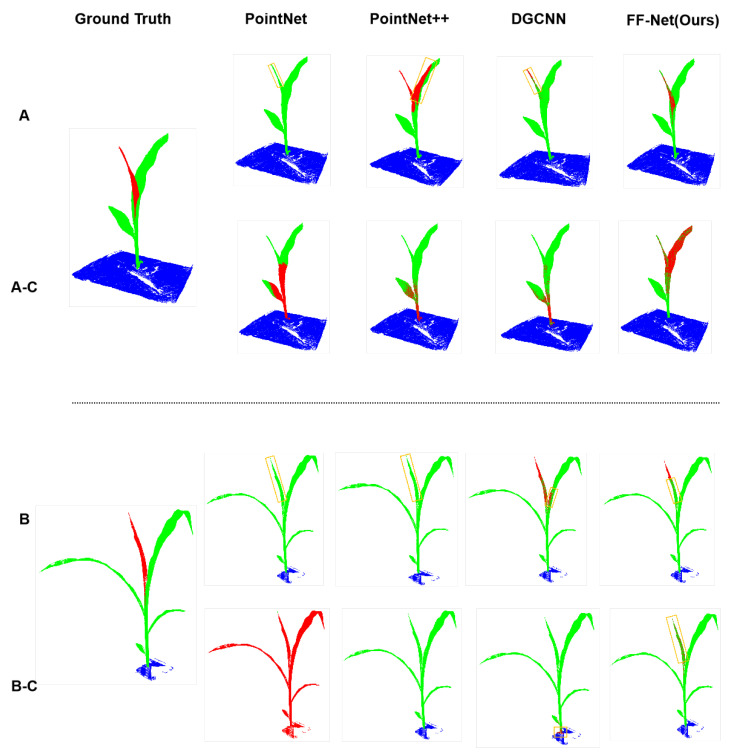
Comparison of semantic segmentation on the maize point cloud between our proposed method and other competitors. The top rows (**A**,**B**) are the segmentation results that were produced by networks trained on the maize dataset, while the bottom rows (**A-C**,**B-C**) are the corresponding cross-validation, i.e., produced by the corresponding networks trained on the tomato dataset.

Visualization: Figure 3 presents the cross-validation results on the maize dataset of the above methods, in which Row A (and B) indicates that the models were trained and evaluated on the maize dataset, while Rows A-C (and B-C) denote that the models were trained on the tomato dataset, but evaluated on the maize dataset. We can see that almost all models failed to obtain precise segmentation results, when trained on a different category of the datasets. PointNet misidentified almost the whole stem as leaves and the bottom leaf as the stem when trained on the tomato dataset, as presented in Rows A-C. For the more complicated maize shown in Rows B-C, almost all points were predicted as the stem class, which was the wrong prediction. As a result, PointNet [25] did not generalize well on the different categories of the datasets. PointNet++ [27] performed slightly better than PointNet [25] in generalizability on the different datasets. As we can see in Rows A-C and B-C of Figure 3, PointNet++ [27] trained on the tomato dataset did not identify the stems of the maize dataset precisely, which performed worse than when it was trained on the maize dataset. DGCNN obtained similar performance to PointNet++ [27] in generalizability to the different datasets.

#### 2.5.2. Training on Maize and Testing on Tomato

Results: The segmentation results of the cross-validation for which the models were trained on the maize dataset and evaluated on the tomato dataset are shown in Table 4. As we can see from the table, all models obtained relatively high IoUs for the ground class (>95%) and leaf class (>90%). However, the IoU for the stem class of all models dropped sharply compared to that of the models trained on the tomato dataset, which was similar to the cross-validation mentioned above. PointNet [25] did not identify any of the stem points of the test dataset when generalized to the tomato dataset from the maize dataset.

**Figure 4:**
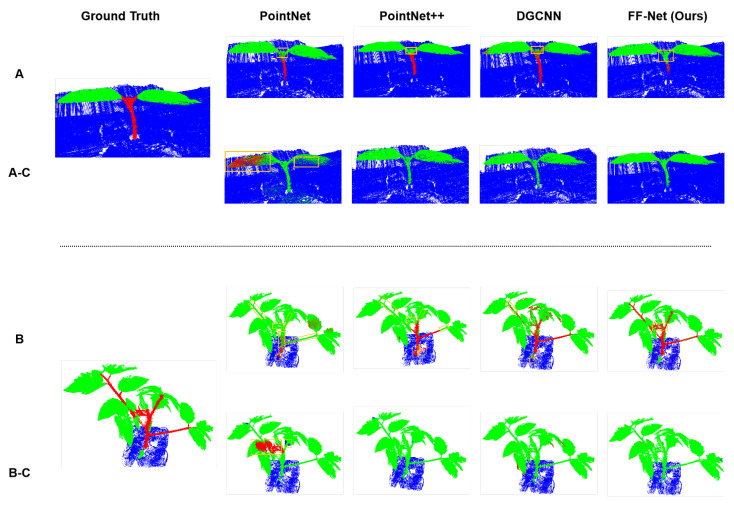
Comparison of semantic segmentation on the tomato point cloud between our proposed methods and other competitors. The top rows (**A**,**B**) are the segmentation results that were produced by the networks trained on tomato dataset, while the bottom rows (**A-C**,**B-C**) are the corresponding cross-validation, i.e., produced by the corresponding networks trained on the maize dataset.

Visualization: Figure 4 shows the cross-validation results on the tomato dataset of all these methods, in which Row A (and B) indicates that the models were trained and evaluated on the tomato dataset, while Rows A-C (and B-C) denote that the models were trained on the maize dataset, but evaluated on the tomato dataset. As we can see, all models almost correctly classified the ground and leaf points, except for PointNet, which misidentified part of the leaves as the stems when trained on the maize dataset. However, all models barely recognized the stem points when generalizing from the maize dataset to the tomato dataset.

## 3. Materials and Methods

### 3.1. Datasets

In this study, we adopted Pheno4D [28] as the experimental dataset. The Pheno4D dataset has a total number of 224 point clouds, which were captured daily from 7 maize and 7 tomato plants after the first sprouts of the plants were observed. The maize dataset was captured for about two weeks and the tomato for about three weeks, which means that all the data were obtained in an early growth stage.

### 3.2. Data Pre-Processing

**Figure 5:**
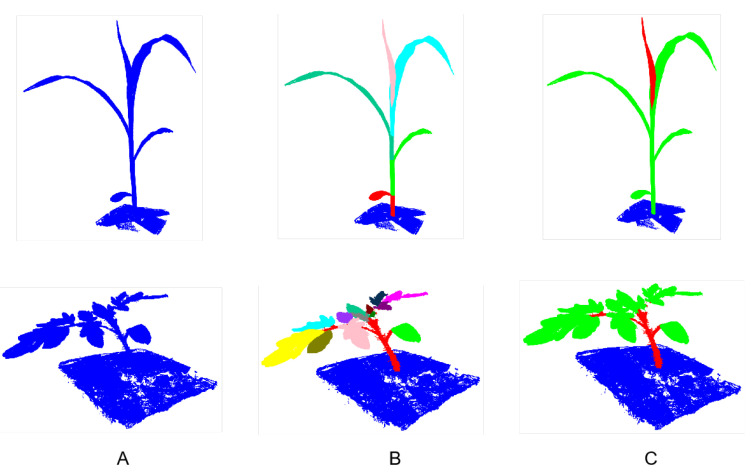
The upper row shows the maize point cloud, and the bottom shows tomato. (**A**): Original point cloud. (**B**): Segmentation with each leaf having a distinct label. (**C**): Segmentation with each category having a distinct label.

The points from Pheno4D are labeled as “ground”, “stem”, or “leaf”, in which the point of the same leaf receives its unique label, making it distinct from the other leaves on the same plant. As the focus of our work was to study plant semantic segmentation, we relabeled all leaf points with the same label. For maize plants, there are two methods to separate the plant point cloud into stems and leaves, namely the leaf collar method and the leaf tip method. To be suitable for subsequent processing of the data, we used the labels derived from the leaf tip method to generate new unified leaf labels. Figure 5 presents the original segmentation and our relabeled semantic segmentation.

We used Plants 1–5 as the training set and Plants 6–7 as the testing set, for both maize and tomato. From Table 5, we can see that the maize plants had 35 point clouds as the training set and 14 as the testing set, while the tomato plants had 55 point clouds as the training set and 22 as the testing set. The distribution of points in the semantic classes is critical to a learning-based network. Table 6 shows the distributions of leaves, stems, and the ground, where the ground accounts for the largest percentage, while stems account for a small proportion. The inhomogeneous distribution of these classes introduces challenges to learning-based methods. In addition, we put all point clouds into a file in the Hierarchical Data format (HDF) for convenience and rapid access.

As plants grow over time, the size of point clouds in different growth stages changes significantly. Table 7 shows the average number of points for a plant cloud captured at different stages. The columns in Table 7 correspond to the stages, from which we can see that the quantitative gap from the lowest to the highest can be more than 2.94 million. The heterogeneous size of different plant point clouds should be taken into account when designing segmentation methods to partition plants into different organs. For constructing experiments for previous point-cloud-learning-based methods, such as PointNet [25], PointNet++ [27], and DGCNN [26], we followed the commonly used strategy for plant point clouds, where a large-scale point cloud is partitioned into fixed-size cubic blocks, each of which is then processed independently by a deep learning network. We also sampled the points in a block at a fixed number of N (2048 was chosen in our experiments). For a fair comparison, we did not drop any point that remained after sampling, so there would exist some blocks with less than 2048 points. After the inference phase, the predictions of blocks from the same plant point cloud were combined to obtain a full segmentation. Our code and relabeled dataset are available at: https://github.com/daojianqingchou/FF-Net, accessed on 8 April 2023.

### 3.3. Methods

#### 3.3.1. Point-Based Methods

Most point-based deep learning methods take as the input the point cloud with a fixed number of points. However, a point cloud captured by LiDAR devices usually contains hundreds of thousands of points. Feeding the entire point cloud to the networks costs a huge amount of memory, which is a challenge to devices such as video cards. The most-straightforward solution to this challenge is down-sampling the point cloud to an acceptable scale with a large rate, which results in a significant loss of geometric details. Some researchers use another strategy, in which a point cloud is partitioned into fixed-size cubic blocks, and then, the block is processed as an independent point cloud in parallel. In these methods, the choice of the block size depends on the scale of the point cloud. If a cubic block is large, the down-sampling of the points in the block to a fixed number of points will introduce a severe loss of geometric information. In turn, the small blocks lead to a large resolution of the point cloud, which does not actually reduce the memory computational cost.

#### 3.3.2. Voxel-Based Methods

**Figure 6:**
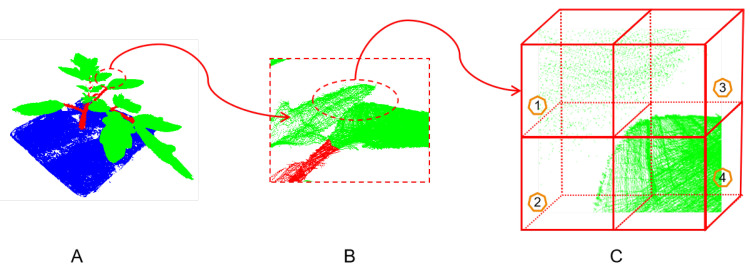
The inhomogeneous distribution of points in different voxels. (**A**): A tomato point cloud. (**B**): Part of the tomato point cloud for A. (**C**): The voxelization of part B, where voxel 4 has more points than voxel 1, 2, and 4.

The voxel-based methods, which are another kind of substitute to process large-scale point cloud, first convert points into many voxels and then apply vanilla 3D convolutions. One of the biggest advantages of this method is that it can maintain the physical properties of point clouds and apply standard convolutional frameworks. This is actually a regular grouping method according to the voxel in which the points reside. Due to the factors such as the size, occlusion, and inhomogeneous distribution of plant points, the voxelized point cloud is sparse and has a highly variable point density throughout the space. Therefore, after grouping through voxelization, voxels of the plant point cloud would contain a variable number of points. As illustrated in Figure 6, where a part of a tomato point cloud is voxelized and enlarged, Voxel 4 has significantly more points than the other three voxels.

#### 3.3.3. Proposed Method

**Figure 7:**
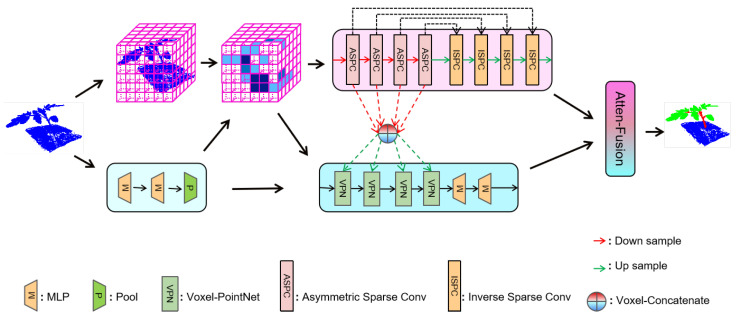
Overall architecture. Here, the top row is the voxel-branch, which captures the local geometric relationship. The middle row is the point-branch, which learns pointwise features and adopts a series of Voxel-PointNet blocks to strengthen the relationships between points within a voxel. The bottom row presents the symbol annotations.

To handle the previously mentioned challenges: (1) the scale of the plant point clouds was changed along with the growth stages; (2) a sub-sampling process was needed to handle the large-scale point cloud for existing methods, and we propose a fusion-based method, which consists of a voxel-branch, a point-branch, and an attention-based fusion method, as illustrated in Figure 7. We give a detailed description of each branch in the following sections.

#### 3.3.4. Voxel-Branch

The voxel-branch partitions a plant point cloud into many equal voxels, each of which contains a different amount of points. Then, a mini-PointNet is employed to extract the features of the points that reside in a voxel, followed by a gathering module to generate a global feature as the feature of the voxel. To fully explore the geometric relationships between the points in a voxel, we devised an adaptive feature extractor, which is formulated as

(3) $$f_{i}=MLPs\left(p_{i}⊕\left(p_{i}-\sum_{j\in V_{i}}p_{j}\right)⊕\left(p_{i}-v_{i}\right)\right),$$

where $p_{i}$ denotes the point coordinates of the *i*-th point, $p_{j}$ indicates the point among the voxels in which the point *i* resides, and $v_{i}$ denotes the border coordinates of the voxel containing *i*. Finally, the concatenation of the three parts (⊕ is the concatenating operator) forms the voxel feature through a linear layer.

#### Asymmetric Sparse Convolution Block

**Figure 8:**
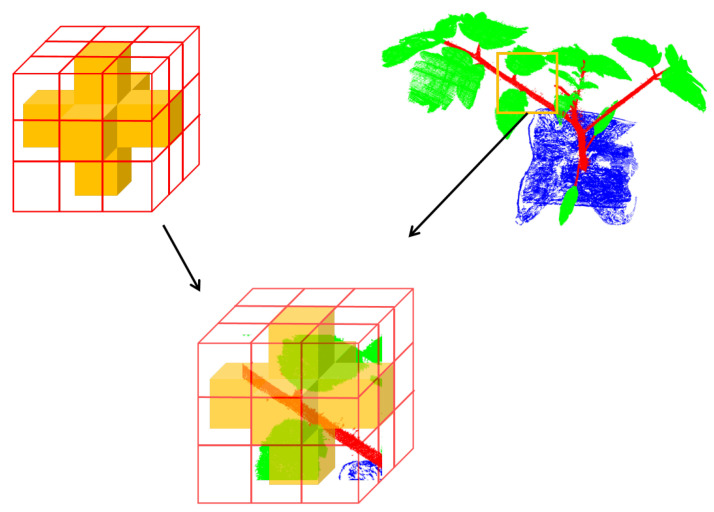
The asymmetric 3D kernel to strengthen the skeleton part of plants.

Due to the heterogeneous distribution of plant organs, the voxels from the plant point clouds are sparse, which is even worse when the resolution is increased. To address this challenge, we adopted a submanifold-based 3D sparse convolutional framework. Inspired by the observation and conclusion in [29], we designed an asymmetric residual block to strengthen the horizontal and vertical kernels, as illustrated in Figure 8, which alleviated the influence of sparsity. Actually, the asymmetric kernel may cover most of the critical parts of the plant structures. Additionally, the designed asymmetrical residual block could save computational cost and memory due to the fewer weights compared with the regular cubic kernel.

#### Inverse Sparse Convolution Block

After a series of asymmetric sparse convolution (ASPC) blocks, the remaining voxels have learned the enriching contextual information between points at different scales. Here, we used a very similar U-net structure for the voxel-branch to connect four inverse sparse convolution (ISPC) blocks to restore the original shape. Skip connections were used to connect the features from the ASPC blocks and ISPC blocks with the same spatial shape.

#### 3.3.5. Point-Branch

As the main objective of the voxel-branch is to extract contextual information at different scales, the points in a voxel still lack specific pointwise features. As a result, points in a voxel share the same voxel label, which could be ambiguous when two different parts of a plant point cloud (e.g., leaf and stem) are partitioned into a voxel.

To address this issue, we designed the point-branch to explore detailed pointwise features. First, we adopted a mini-PointNet for the raw inputs to produce learned points’ features, which were fed into the voxel partitioning module. Then, the features of the points were partitioned into voxels according to their coordinates, followed by an aggregating operation to generate the voxel features. It is worth noting that the voxel partition actually acts as the neighbor area searching module, which is similar to the K-nearest neighbor (KNN) searching strategy, but more efficient. The voxelwise features and pointwise features output from mini-PointNet were fed into a series of Voxel-PointNet (VPN) blocks to produce more particular pointwise features with contextual information from different scales. Finally, a feature fusion module based on an attention mechanism (Atten-Fusion) was devised to fuse the features from both branches. We detail the VPN and Atten-Fusion methods in the following sections.

#### Voxel-PointNet Block

PointNet [25] and its upgraded version PointNet++ [27] have an excellent capacity to extract point features and neighboring point features, respectively. However, the large computational and memory cost of the K-nearest neighbor searching methods prevent both PointNet and PointNet++ from being extended to large-scale point clouds. In this work, we propose a voxel-based PointNet, which aggregates point features by considering the voxels produced by the voxel-branch as neighboring regions. The VPN is an efficient strategy to easily obtain multi-scale hierarchical features by reusing the ASPC block automatically to down-sample the voxel scale.

#### Attention-Based Fusion Module

**Figure 9:**
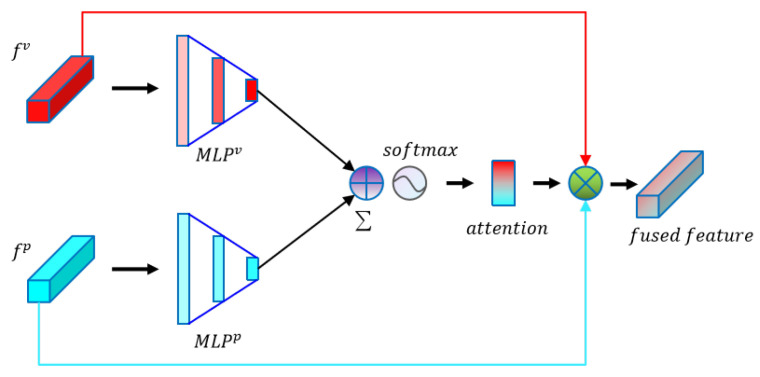
Attention-based feature fusion module.

For each point, it eventually obtains point features and voxel features from the point-branch and voxel-branch, respectively. It is an essential task to fuse useful features from both branches together under the interference of massive useless information. In general, the two features are summed up or concatenated to produce fused features, but both approaches suffer from a large number of non-informative features, which can be formulated as

(4) $$\begin{array}{cc} \tilde{f} & =concatenate(f_{1},\ldots ,f_{N}), \end{array}$$

(5) $$\begin{array}{cc} \tilde{f} & =\sum_{i=1}^{N}f_{i}, \end{array}$$

where $\tilde{f}$ is the fused features. Inspired by the attention mechanism, which can focus on useful information by measuring the importance of each feature channel, we designed an attention-based fusion (AF) module to filter useless information. Our AF module calculates two attention score vectors for both branch features of each point and sums the vectors followed by a softmax function. The calculation of the attention score is formulated as follows:

(6) $$\alpha_{i}=softmax\left(\sum_{b\in \{v,p\}}MLP_{b}\left(f_{i}^{b}\right)\right),$$

where *b* denotes the branch type, either voxel or point; $MLP_{b}$ here also corresponds to the branch type. The fused point features are calculated by summing the product of point features from both branches and their corresponding attention scores, formulated as:

(7) $$\tilde{f_{i}}=\left[f_{i}^{v},f_{i}^{p}\right]\times \alpha_{i},$$

where $\tilde{f_{i}}$ denotes the fused features of point *i* and $f_{i}^{v}$ and $f_{i}^{p}$ indicate the point features from the voxel-branch and point-branch, respectively. Figure 9 illustrates the details of our proposed attention-based feature fusion module.

#### Loss Function

The imbalanced distribution of different classes introduces a challenge to the training of neural networks. Take, for example, the fact that, in the point cloud of maize or tomato, the appearance of stems is much less than the leaves and ground. This issue biases the neural network towards the classes that appear more in the dataset and limits the performance of the network.

To address the imbalanced distribution issue, we followed the existing methods [30,31] and added more weights to the less-represented class by using the weighted cross-entropy loss. Our total loss function was composed of two parts, namely voxel-loss and point-loss. For the voxel-loss, we used a combination of the weighted cross-entropy loss and Lovász-softmax loss to maximize the intersection over union (IoU), which can be formulated as

(8) $$\begin{array}{cc} Loss_{v} & =-\sum_{i}\alpha_{i}p\left(y_{i}\right)log\left(p\left(\tilde{y_{i}}\right)\right)+lovasz\left(y,\tilde{y}\right) \end{array}$$

(9) $$\begin{array}{cc} \alpha_{i} & =\frac{1}{\sqrt{f_{i}}}, \end{array}$$

where $f_{i}$ indicates the frequency of the *i*-th class and $y_{i}$ and $\tilde{y_{i}}$ denote the true and predicted class, respectively. For the point-loss, only the weighted cross-entropy loss was adopted to supervise the training. The total loss can be formulated as

(10) $$Loss=Loss_{v}+Loss_{p}.$$

## 4. Discussion

### 4.1. Efficiency of the Methods

The average latency of these methods on a 3D plant point cloud is presented in Table 8. We can see that the same method had a higher latency on the tomato dataset than on the maize dataset, as a tomato point cloud had a more complicated structure and more points than a maize point cloud. PointNet [25] had the lowest latency on both the maize and tomato datasets, while PointNet++ [27] and DGCNN [26] had the highest and second-highest latency, respectively. This was because both PointNet++ [27] and DGCNN [26] adopted KNN as the neighbor area search strategy, which has a $O(n^{2})$ time complexity. PointNet is a pure MLP-based method without any neighbor searching operations, and our method relies on voxelization to search the neighborhood area, which has a O(n) time complexity. Our proposed method, called FF-Net, employs two branches, i.e., the voxel-branch and the point-branch, to overcome this problem. The voxel-branch takes full advantage of the 3D sparse convolution to extract spatial features efficiently, and the point-branch adopts mini-PointNet to learn the point features within a voxel, completing the pointwise features. This is one of the reasons why our method had higher efficiency, but lower latency than the other competitors. Another reason why our proposed method was more efficient was that mini-PointNet extracted point features only within the voxel in which the point resides, which saves time in searching the local areas.

### 4.2. Effectiveness of the Methods

The experimental results showed that our proposed method outperformed the competitors in terms of the mIoU and almost the IoU in all classes for the maize and tomato datasets. The main reason was that our method took as the input the original points without any down-sampling, which enabled the model to learn richer features. The semantic segmentation task of point clouds depends not only on pointwise features, but also on the relationships between the neighboring points. Another reason why our proposed method was more effective than the other competitors was that our model fuses the point features from point-branch and voxel-branch through an attention-based module, which could filter the useless features to preserve more useful information based on trainable attention weights. In addition, we conducted cross-validation experiments to evaluate the generalization ability of these methods. The experimental results showed that the methods learning relationships between points (e.g., PointNet++ [27], DGCNNN [26], and FF-Net) performed better than the method only learning point features (e.g., PointNet [25]). It is worth noting that our method achieved the best mIoU on the maize dataset, while DGCNN performed better on the tomato dataset in the cross-validation experiments. This was because DGCNN is a strongly graph-based method that can fully explore the edge relationships between the center and neighboring points. However, the KNN-based neighbor search method adopted by DGCNN is time-consuming, which impeded its application to the high-resolution point clouds.

## 5. Conclusions

This work focused on the semantic segmentation of high-resolution 3D plant point clouds. To this end, we relabeled the large-scale plant dataset Pheno4D [28] according to the semantic categories and generated a Hierarchical Data Format (HDF) file for accelerating access. Then, a feature-fusion-based method named FF-Net was proposed to segment the point cloud by two branches, namely the point-branch and voxel-branch. The experimental results showed that the proposed method outperformed three widely used methods on both the maize dataset and the tomato dataset. Furthermore, we explored the generalization capabilities of these methods, and our method achieved competitive results. We hope that this work will provide a novel idea for the semantic segmentation of the high-resolution 3D plant point clouds. In the future, we will continue improving the recognition of small classes and enhancing the generalization ability of the model.

## Figures and Tables

**Table 1:** Semantic segmentation results on maize datasets.

Network	mIoU	Ground	Stem	Leaf
PointNet [25]	61.59	98.16	3.40	83.21
PointNet++ [27]	74.07	99.25	35.37	87.58
DGCNN [26]	75.60	99.56	38.37	88.87
FF-Net	80.95	99.58	52.76	90.53

**Table 2:** Semantic segmentation results on tomato point cloud datasets.

Network	mIoU	Ground	Stem	Leaf
PointNet [25]	73.74	99.61	28.35	93.25
PointNet++ [27]	81.03	99.41	48.78	94.91
DGCNN [26]	83.96	99.67	56.73	95.49
FF-Net	86.65	99.56	64.17	96.21

**Table 3:** Semantic segmentation results trained on the tomato point cloud datasets and evaluated on the maize datasets.

Network	mIoU	Ground	Stem	Leaf
PointNet [25]	58.68	99.22	0.35	76.47
PointNet++ [27]	63.37	97.80	2.85	89.45
DGCNN [26]	60.3	98.93	0.69	81.28
FF-Net	63.95	95.62	6.71	89.52

**Table 4:** Semantic segmentation results trained on the maize point cloud datasets and evaluated on the tomato datasets.

Network	mIoU	Ground	Stem	Leaf
PointNet [25]	63.14	98.93	0.0	90.49
PointNet++	63.37	97.80	2.85	89.45
DGCNN	64.38	99.68	2.75	90.72
FF-Net	63.85	96.59	3.83	91.12

**Table 5:** Amount of training and testing datasets.

Category	Training Set	Test Set
Maize	35	14
Tomato	55	22

**Table 6:** Distribution of classes in the training set and testing set.

Category	Training Set (%)			Testing Set (%)		
	Ground	Stem	Leaf	Ground	Stem	Leaf
Maize	50.03	5.50	44.47	48.56	7.40	44.04
Tomato	49.35	4.56	46.09	50.73	4.32	44.95

**Table 7:** The average number of points (millions) for plant point clouds captured at different stages.

	**1**	**2**	**3**	**4**	**5**	**6**	**7**	**8**	**9**	**10**	**11**
Maize	-	-	-	-	1.53	1.57	0.74	0.82	1.02	1.25	1.33
Tomato	2.01	2.76	2.53	2.34	2.94	2.16	2.34	1.90	1.88	3.68	3.64

**Table 8:** The average latency (seconds) of the methods on semantic segmentation tasks.

Dataset	FF-Net	PointNet [25]	PointNet++ [27]	DGCNN [26]
Maize	5.57	4.42	96.85	9.14
Tomato	30.18	27	244.18	40.27

## Data Availability

Not applicable.
